# Assessment of Violence during COVID-19 among Reproductive Age Women in Arsi Zone, South East Ethiopia

**DOI:** 10.1155/2024/2044708

**Published:** 2024-02-27

**Authors:** Gebi Agero, Kelil Haji Bedane, Mesfin Tafa Segni, Abdene Weya Kaso, Aman Nebi Ayato, Dida Batu Bedada, Akalu Ermias Mieso

**Affiliations:** ^1^Department of Public Health, College of Health Science, Arsi University, Asella, Ethiopia; ^2^Department of Anatomy, College of Health Science, Arsi University, Asella, Ethiopia; ^3^Department of Medical Laboratory Science, College of Health Science, Arsi University, Asella, Ethiopia; ^4^Department of Surgery, College of Health Science, Arsi University, Asella, Ethiopia

## Abstract

**Background:**

Preventive measures, like staying at home during lockdown, are mandatory during the COVID-19 pandemic. Particularly as a result of staying at home, violence against women is beginning to increase in correlation with these measures. Therefore, the purpose of this study was to assess the prevalence of violence against women of reproductive age during the COVID-19 pandemic in the Arsi Zone.

**Methods:**

A community-based cross-sectional study design was employed from February 15 to March 30, 2021. A multistage sampling technique was used to recruit 1458 women aged 15-49 years old. Data entry was performed using Epi info-7 and exported to SPSS version 25 for analysis. A logistic regression analysis was employed to identify factors associated with violence against women at *p* value <0.05 and AOR values with 95% CI.

**Results:**

The prevalence of violence against women during COVID-19 was 51.1% (95% CI 48.5-53.7%). Psychological violence (31.8%) and controlling behavior violence (29.3%) were the leading types of violence followed by economic (20.2%) and sexual violence (15.6%). Respondents who had monthly income of <1000 birr (AOR = 1.72; 95% CI, 1.18, 2.51), 1001-2000 birr (AOR = 2.22; 95% CI, 1.51, 3.27), 2001-3000 birr (AOR = 1.91; 95% CI, 1.26, 2.91), and 3001-4000 birr (AOR = 2.03; 95% CI, 1.31, 3.14), quarreled with their partner's family (AOR = 3.36; 95% CI: 2.14–5.30), witnessed chilhood family violence (AOR = 2.34; 95% CI: 1.81–3.02), and decisions made on the household issue by husband only (AOR = 2.62; 95% CI: 2.01–3.41) or wife only (AOR = 1.99; 95% CI: 1.33–2.98) were significantly associated with violence against women. In addition, we found that participants whose partners cannot read and write (AOR = 2.63; 95% CI: 1.19– 5.81), drink alcohol (AOR = 2.78; 95% CI: 2.10–3.76), chew chat (AOR = 3.27; 95% CI: 2.21–4.85), ever fighting or aggressive with other men (AOR = 2.73; 95% CI: 1.51–4.95), and partners' families taking part in the decision making (AOR = 2.32; 95% CI: 1.49–3.62) were also associated with violence against women.

**Conclusions:**

One in every two women was the victim of any form of violence in the study area. Hence, empowering women's economic status and enhancing community-based health education for males on behavioral lifestyle modification were required to prevent violence against women.

## 1. Background

Any act of gender-based violence against women (VAW) that causes or is likely to cause bodily, sexual, or emotional injury or suffering to women is considered VAW. This includes coercion, threats of such actions, and arbitrarily denying a woman her freedom, whether the abuse takes place in public or secret. Sexual and intimate partner violence (IPV) are the main causes of public health issues, which are a violation of human rights and discriminate against women [1–3].

Violence against women is an epidemic-sized issue that harms women for the rest of their lives. For women and girls, it can have a variety of short-term to long-term medical, sexual, and emotional effects, including death. It has severe consequences, including higher health care and legal costs, lost productivity, and overall development. It also negatively impacts women's general well-being and keeps them from fully engaging in society, as well as the economic and social health of their families and communities [4–6].

In addition, IPV relationships and sexual assault have significant social and financial costs that have an impact on the entire community. Women may experience loneliness, be unable to work, lose their jobs, be unable to participate in regular activities, and have reduced capacity to take care of their children and themselves. Gender is a significant factor in humanitarian crises, impacting not only psychosocial outcomes but also health, socioeconomic, and political realities. The general health, social, and economic standing of the population, particularly that of women, is particularly vulnerable as humanitarian crises worsen [7–9].

Globally, either physical or sexual abuse by an intimate partner, or both, has been experienced by 35.6% of women. According to regional estimates, the frequency of sexual violence against people without partners and IPV together ranges from 27.2% in Europe to 45.6% in Africa. According to an analysis by the World Health Organization, before the COVID-19 pandemic, rates of IPV in Africa were as low as 19.5% in Namibia and as high as 53.7% in Ethiopia [10–13].

Population-based violence against women in Ethiopia varies depending on the perpetrator. Studies among women in rural communities in Kersa, Butajira, and the Awi Zone found that rates of violence by intimate partners ranged from 19.6% to 78% [10, 14, 15]. In addition, even though data was sparse in the era of COVID-19 in Ethiopia regarding VAW, 22.4% of married women in the northern part of Ethiopia, Dessie town, were victims of violence by their partner [16]. Moreover, the extent of VAW in the Oromia region is variable based on the status of the woman; as high as 59% to 64.6% of pregnant women have experienced violence by an intimate partner in the East Shoa zone and Bale zone, respectively [17, 18]. Factors like lower levels of education, exposure to child maltreatment, witnessing chilhood family violence, alcohol consumption, accepting attitudes and norms that accept violence, gender inequality, and male control/controlling behavior over women (i.e. unequal power in intimate relationships) were reported to be risk factors for both women's experience and men's perpetration of partner violence [10, 11, 14, 15, 16, 19–21]. Despite the government's effort to reduce violence against women, the COVID-19 pandemic had a substantial impact on women due to the lockdown orders and longer duration of contact with their partner or nonpartners which exposed them to psychological abuse than before the pandemic [22]. In addition, emerging statistics and reports from frontline workers have demonstrated that all forms of violence against women and girls, including domestic abuse, have escalated since the COVID-19 outbreak [7, 23, 24]. However, in Ethiopia, including the study area, there is little information on the prevalence of violence against women during the COVID-19 pandemic due to lockdown measures. Thus, the lack of concrete evidence on the prevalence of violence against women of reproductive age poses difficulty for health providers and local and national policymakers to design preventive measures. Having a better understanding of how the COVID-19 lockdown affects violence against women could help with the development of preventative programs both during and after the pandemic to achieve Sustainable Development Goal 5 by the end of 2030. Thus, this study is aimed at assessing the prevalence of violence and associated factors among women aged 15-49 years during the COVID-19 pandemic in Arsi Zone, South Central Ethiopia.

## 2. Methods and Materials

### 2.1. Study Area, Period, and Design

A community-based cross-sectional study design was conducted in the Arsi Zone, South Central Ethiopia, from February 15 to March 30, 2021. Administratively, Arsi Zone is divided into 26 districts and 2 administrative towns (Asella and Bokoji), with an estimated area of 23,679.7 km^2^. Based on the 2007 Housing and Population Census, the total population of Arsi Zone is projected to be 3,459,322 million in 2017, of which 2,965,947 of the population was estimated to be rural residents and 1,730,722 were estimated to be females [25]. The average altitude ranges from 1700 to 4000 meters above sea level. The average temperature varies from 10 to 24°C. Agriculture is the main economic source of the population. Barley, wheat, teff, sorghum, and onion are the major agricultural products produced in the area.

### 2.2. Sample Size Determination and Sampling Procedure

The final sample size was calculated using a single population proportion formula with assumptions, 2.67% calculated margin of error, 95% confidence intervals (CI), design effect of 1.5, and proportion of VAW (19.6%) from a study conducted in Kersa Community [15]. The calculated final sample size for this study was 1466 considering the 15% nonresponse rate. Multistage sampling techniques were used to employ the study participants. In the first sampling method, six towns (Asella, Abomsa, Robe, Dera, Bokoji, and Kersa) were purposely selected as there are larger towns in the zone that have implemented lockdowns during the state of emergency. The sample size was proportionally allocated to the number of women aged 15-49 years in the town. Study households were selected from each kebele by systematic sampling. Subsequent households were selected based on the household interval in the kebeles, which was determined by dividing the total number of households by the required sample size. In each household, one woman aged between 15 and 49 years was interviewed. In a household where there is more than one eligible woman, a woman with a husband was interviewed, and for two women with a husband, the lottery method was used to select one of them when there were no eligible women in the selected household, and the next household was visited.

### 2.3. Variables of the Study

#### 2.3.1. Dependent Variable: Violence against Women

#### 2.3.2. Independent Variables

Sociodemographic characteristics of women are age, ethnicity, occupation, marital status, educational status, average monthly income, religion, and family size.

Behavioral-related factors of women are witnessing child violence, social norm of husband to beat wife, decisions made on the household issue, and history of family violence.

Sociodemographic characteristics of nonpartner/partner are age, ethnicity, occupation, marital status, educational status, average monthly income, religion, and family size.

Nonpartner/partner behavioral-related factors are smoking cigarettes, drinking alcohol, chewing chat, aggressively fighting with other men, and partner's family involved in decision-making.

### 2.4. Data Collection Procedure and Quality Management

An interviewer-administered structured questionnaire was adapted from previous studies and WHO standard questions for the assessment of VAW [10]. A questionnaire was prepared in English and then translated into the local language (i.e., Afan Oromo), and then, it was translated back to English to check for its consistency. Eight data collectors who have master's degrees in nursing, midwifery, and public health were recruited for data collection. Before data collection, questionnaire was pretested on 5% of the total sample outside of the study area, then based on the result of pretest, amendment was made to the questionnaire accordingly. Two days of training were provided to the data collectors and supervisors on the purpose of the study, principles, and ethical considerations of the data collection process. The local language (Afan Oromo) version of the questionnaire was used for data collection. The supervisors made a day-to-day on-site supervision whereas the principal investigator checked each questionnaire daily for completeness and consistency.

### 2.5. Data Processing and Analysis

The collected data were entered into Epi.info version 7.2 and imported to Statistical Package for Social Science (SPSS) version 25 software for analysis. Descriptive statistics such as frequency, percentage, mean with standard deviation, and median with interquartile were used to describe sociodemographic and other relevant variables. A multivariate logistic regression model was used to determine the association between the different variables and outcome variables. Independent variables that were significant with a *p* value less than 0.25 in bivariate logistic regression analysis were included in a multivariate analysis. A *p*-value of less than or equal to 0.05 and an adjusted odds ratio (AOR) with 95% CI were used to declare factors associated with violence against women. The fitness of the model was checked by using the Hosmer and Lemeshow goodness-of-fit test.

## 3. Results

### 3.1. Sociodemographic and Economic Characteristics of the Participants

Out of the 1466 study participants, 1458 participated in the study with a response rate of 99.5%. Out of the respondents, 868 (59.5%) of them were aged less than 30 years of age, and the median age of the respondents was 28 years with an interquartile range (IQR) of 11 years. The majority of the respondents (820, 56.2%) were Orthodox religious followers, and 510 (35%) attended secondary education (Table 1).

### 3.2. Sociodemographic and Economic Characteristics of the Partner/Nonpartners

Out of 1458 respondents, 590 (40.5%) had partner/nonpartners aged 31-40 years, and 69.9% of their regular or nonregular partners have a secondary and above level of education. Three hundred forty-three (23.5%) of the women had a partner with an alcohol-drinking habit, while a sizable proportion (15.5%) of the respondents had partners with chat chewing behavior. In addition, 114 (7.8%) of the women had a partner that aggressively fought with other men, whereas around 143 (9.8%) of the participants had a partner that quarreled with their partner's family (Table 2).

### 3.3. Types of Violence Perpetrated among Reproductive-Aged Women

In the study area, a total of 745 (51.1%, 95% CI: 48.5-53.7%) women were victims of any form of violence. Two hundred twenty-four (15.4%, 95% CI: 13.6, 17.3%) of the women were physically violated by their regular or nonregular partners during COVID-19 while 228 (15.6%, 95% CI: 13.8, 17.6%) of them were also victims of sexual violence during COVID 19. Besides, 31.8% (95% CI: 29.4, 34.2%) and 29.3% (95% CI: 27.0, 31.8%) were psychological and controlling behavior victims, respectively. The most commonly cited physical violence attribute was being slapped or thrown something that could harm while controlling behavior, violence, acting jealous, and getting angry if she speaks with another man were the most frequently cited attributes among respondents (Table 3).

### 3.4. Sociodemographic and Behavioral Characteristics of the Participants Associated with Violence against Women during COVID-19

A total of twelve respondent's sociodemographic characteristics were evaluated to be a candidate for multivariate analysis with a *p* value of <0.25, and five variables (age of the respondent, ethnicity, religion, occupation, and number of family members) were excluded from the final model since their *p* value was greater than 0.25. The likelihood of violence was 1.72 and 2.22 times higher among respondents who had monthly income of less than 1000 birr and 1001-2000 birr compared to those who had monthly income of greater than 5000 birr (AOR = 1.72; 95% CI: 1.18, 2.51 and AOR = 2.22; 95% CI: 1.51, 3.27), respectively. In addition, women who had monthly income of 2001-3000 birr and 3001-4000 birr were 1.91 and 2.03 more likely to experience violence compared to those who had monthly income of greater than 5000 birr (AOR = 1.91; 95% CI: 1.26, 2.91 and AOR = 2.03; 95% CI: 1.31, 3.14), respectively. Moreover, participants who reported decisions made on household issue by husband or wife only were almost three and two times more likely to experience violence compared to those who made joint decisions (AOR = 2.62; 95% CI, 2.01, 3.41) and (AOR=1.99; 95% CI, 1.33, 2.98), respectively. The odds of violence were higher among respondents who quarreled with their partner's family compared to their counterparts (AOR = 3.36; 95% CI: 2.14, 5.30). Moreover, the odds of violence were higher among respondents who witnessed violence as a child between parents compared to their counterparts (AOR = 2.34; 95% CI: 1.81, 3.02) (Table 4).

### 3.5. Sociodemographic and Behavioral Characteristics of the Partners/Nonpartners Associated with Violence against Women

In the bivariate analysis, the partner/nonpartner-related factors that showed association with the outcome variable were age, partner/nonpartner level of education, partner/nonpartner occupation, partner/nonpartner behavior factors (chewing chat, smoking cigarettes, drinking alcohol, and aggressively fight with other men), and partner's family involved in decision-making. However, in multivariable logistic regression, only variables such as partner/nonpartner level of education, partner/nonpartner behavior factors (chewing chat, drinking alcohol, and aggressively fighting with other men), and partner's family involvement in decision-making had a significant association with violence against women. We found that participants whose partners cannot read and write were almost three (AOR = 2.63; 95% CI: 1.19, 5.81) times more likely to face violence than those whose partners had a diploma and above educational status. In addition, respondents whose partners drank alcohol and chewed chat were almost 3 times more likely to face violence compared to their counterparts (AOR = 2.78; 95% CI: 2.10, 3.76 and AOR = 3.27; 95% CI: 2.21, 4.85), respectively. The odds of violence among women were almost three (AOR = 2.73; 95% CI: 1.51, 4.95) times higher for women whose partners/nonpartners were ever fighting or aggressive with other men compared to their counterparts. Furthermore, the odds of violence were almost two (AOR = 2.32; 95% CI: 1.49, 3.62) times higher among women whose non partner/partners' families were involved in the decision making compared to their counterparts (Table 5).

## 4. Discussions

This study assessed the prevalence of violence against women and associated factors in the Arsi Zone. The study revealed that the prevalence of violence against women was 51.1% (95% CI: 48.5-53.7%). This finding is lower than the studies conducted in Bale zone (59%) [17] and Gedo woreda (64.6%) [18]. The variation could be possibly related to the type of population studied and the time of the study. The two studies (i.e., Bale zone and Gedo woreda) were among pregnant women. Another possible variation in the difference might be that the risk of violence could be higher among susceptible population groups like pregnant women in the Bale and Gedo studies, whereas the current study was among general women aged 15-49 years of age.

The prevalence of violence in our findings is higher than the studies conducted during COVID-19 in Australia (4.2%) [26], Tunisia (14.8%) [27], Dessie (22.4%) [16], Debre Birhan (19%) [28], Gurage Zone (24.11%) [29], and Gonder town (42.05%) [30]. The possible reason for the variation might be related to the method of violence assessment (the study conducted in Australia used an online method), study period, and the difference in the population studied; the reports from studies were from married women, and they were conducted in one area, town, or woreda among school girls.

Among the types of violence perpetrated against reproductive age women in the study area, psychological violence (31.8%) was found to be the leading type, followed by controlling economic, sexual, and physical violence. This finding is higher than the studies conducted in Australia (11.6%) [26], Dessie town (20%) [16], and Debre Birhan (19.9%) [28]. The possible reason for the higher proportion could be related to the time when the study was conducted; following the lockdown, higher numbers of individuals were forced to stay at home, and harassment and psychological abuse were more likely to occur during tension times. Women who are already in an abusive relationship are more likely to be harassed if there is a longer duration of contact with their partner or nonpartner. There was also a report indicating that women who were psychologically abused before the pandemic were more likely to be abused during the pandemic [27].

In this study, respondents' monthly income was significantly associated with higher odds of violence against women. This finding is supported by findings from previous studies [14, 17]. The possible reason could be that the average monthly income is low; the need to have daily expenses and cover these costs could be a problem that will raise tension between the partner and the woman. This variation can also be partly answered from this study as the odds of violence are as high as three times the fold for women who decide household issues either by themselves or by their husband/partner compared to a joint decision.

In this study, decisions made either by husband or wife only on household issues were associated with violence against women. Besides, women who have witnessed violence among parents as a child were more than two times more likely to encounter violence compared to their counterparts. This figure is in agreement with reports from other parts of the country [17, 26, 30]. This could be related to accepting a partner's aggressive behavior as normal in a society where men's muscularity is accepted as normal.

Women who have partners who are aggressive or ever fight with other men have 2.7 times higher odds of violence compared to their counterparts. This finding is in line with a study conducted in the Bale zone [17]. The possible reason could be that men's muscularity is a predictor of violence either at home or outside.

Partner's level of literacy was also associated with VAW in this study. The odds of VAW whose partners are unable to read and write were more than 2.5 times higher compared to those women's partners whose level of education is tertiary (diploma and above). This is in agreement with reports from other parts of the country that were conducted before and after the pandemic [18, 29]. This could be related to the ease of communication among partners; as those who have some form of level of education are more likely to engage in dialogue than more aggressive behavior to solve the issues that can arise between them.

The likelihood of violence was almost three times higher among women whose partners chewed chat compared to their counterparts. Women whose partners drink alcohol were 2.78 times more likely to be a victim of violence compared to those partners who do not drink alcohol. This finding is supported by reports from other parts of the world [9, 15, 17]. The possible reason could be that alcohol consumption can raise levels of aggressiveness, lead to misunderstanding of verbal or nonverbal cues, and encourage risk-taking behavior which might be a source of dispute in relationships. Our studies may have a large sample size and can be internally and externally valid to the larger population as the representative sample was obtained. However, it has the following limitations. First, because of the nature of the study design itself, it is hard to judge whether all associations are causes. Second, data were collected from women regarding their partner/nonpartner, and social desirability bias may occur; however, we have tried to establish rapport during data collection and confidentiality granted as the participants will not report only positive behavior.

## 5. Conclusion

One in every two women was the victim of any form of violence in the study area. Womens' monthly income, decision made on household issues by husband or wife only, partner's substance use, education level, aggressiveness behaviour, and families' involvement in decision-making were factors associated with violence against women. Hence, empowering women's economic status and enhancing community-based health education for males on behavioral lifestyle modification are required to prevent violence against women.

## Figures and Tables

**Table 1:** Sociodemographic characteristics of women aged 15-49 years old in Arsi Zone, 2021.

Variables	Characteristics	Frequency (%)
Age	15-19	80 (5.5)
	20-24	328 (22.5)
	25-29	460 (31.6)
	30-34	212 (14.5)
	35-39	214 (14.7)
	40-44	89 (6.1)
	>=45	75 (5.1)

Religion	Muslim	499 (34.2)
	Orthodox	820 (56.2)
	Protestant and others	139 (9.5)

Marital status	Single	54 (3.7)
	Have a regular partner but living apart	170 (11.7)
	Married and lived with a partner	1154 (79.1)
	Others (divorced, widowed)	80 (5.5)

Ethnicity	Oromo	967 (66.3)
	Amhara	394 (27)
	Tigre and others	97 (6.7)

Educational status	Unable to read and write	119 (8.2)
	Able to read and write	46 (3.2)
	Primary (1–8)	509 (34.9)
	Secondary (9–12)	510 (35.0)
	Tertiary (diploma and above)	274 (18.8)

Average monthly income	0-1000	395 (27.1)
	1001-2000	282 (19.3)
	2001-3000	193 (13.2)
	3001-4000	153 (10.5)
	4001-5000	138 (9.5)
	>5000	297 (20.4)

Live with	Alone	67 (4.6)
	With a partner (not married)	23 (1.6)
	With your husband	1154 (79.1)
	With husband family	75 (5.1)
	With your family	92 (6.3)
	Others	47 (3.2)

Decisions made on the household issue	Husband	464 (31.8)
	Wife	185 (12.7)
	Jointly	809 (55.5)

**Table 2:** Sociodemographic and behavioral factors of partners/nonpartners of women aged 15-49 years old in Arsi Zone, 2021.

Variables	Characteristics	Frequency (%)
Age	20-30	526 (36.1)
	31-40	590 (40.5)
	41-50	223 (15.3)
	>50	119 (8.2)

Occupation of your partner/nonpartner	Student	37 (2.5)
	Farmer	177 (12.1)
	Employer	577 (39.6)
	Merchant	333 (22.8)
	Others	334 (22.9)

Educational status	Unable to read & write	48 (3.3)
	Read and write	52 (3.6)
	Primary (1–8)	338 (23.2)
	Secondary (9–12)	509 (34.9)
	Tertiary (diploma & above)	511 (35.0)

Drink alcohol	Yes	343 (23.5)
	No	1115 (76.5)

Chew chat	Yes	226 (15.5)
	No	1232 (84.5)

Smoke cigarette	Yes	76 (5.2)
	No	1382 (94.8)

Ever fight (physically aggressive) with other men	Yes	114 (7.8)
	No	1344 (92.2)

Partner's family involved in decisions	Yes	157 (10.8)
	No	1301 (89.2)

Quarreled with partner's family	Yes	143 (9.8)
	No	1315 (90.2)

**Table 3:** Types of violence perpetrated among women aged 15-49 years in Arsi Zone, 2021.

Variables	VAW during COVID-19	
Partner/husband	Yes (%)	No (%)
Physical violence		
Slapped or thrown something that could harm	217 (14.9)	1241 (85.1)
Pushed or shoved the hair	130 (8.9)	1328 (91.1)
Hit with his fist or with something else that could hurt	131 (9)	1327 (91)
Kicked, dragged, or beat	106 (7.3)	1352 (92.7)
Choked or burnt on purpose	57 (3.9)	1401 (96.1)
Threatened to use or used a gun, knife, or other weapons	67 (4.6)	1391 (95.4)
Sexual violence		
Forced to have sexual intercourse without interest	216 (14.8)	1242 (85.2)
Ever have sexual intercourse you did not want (what partner might do)	139 (9.5)	1319 (90.5)
Ever forced to do something sexual that is degrading or humiliating	51 (3.5)	1407 (96.5)
Psychological violence		
Partner/nonpartner ever insulted or made feel bad about yourself	464 (31.8)	964 (69.2)
Belittled or humiliated in front of other people	221 (15.2)	1237 (84.8)
Done things to scare or intimidate on purpose (yelling and smashing things)	147 (10.1)	1311 (89.9)
Threatened to hurt you or someone you care about	116 (8)	1342 (92)
Controlling behavior violence		
Tried to keep from seeing your friends	248 (17)	1210 (83)
Tried to restrict contact with the family of birth	173 (11.9)	1285 (88.1)
Insisted on knowing where you are at all times	269 (18.4)	1189 (81.6)
Acted jealous & get angry if you speak with another man	362 (24.8)	1096 (75.2)
Often been suspicious that you are unfaithful	214 (14.7)	1244 (85.3)
Economic violence		
Taken your earnings or savings from you against your will	185 (12.7)	1273 (87.3)
Refused to give money for household expenses, when he had the money for other things	231 (15.8)	1227 (84.2)

**Table 4:** Respondent's sociodemographic and behavioral characteristics associated with violence against women during COVID-19 in Arsi Zone, 2021.

Variables	Characteristics	VAW during COVID-19			
		Yes (%)	No (%)	COR 95% CI	AOR 95% CI
Marital status	Single	30 (55.6)	24 (44.4)	1	1
	Have a partner but living apart	76 (44.7)	94 (55.3)	0.64 (0.35, 1.20)	0.73 (0.38, 1.40)
	Married & lived with a partner	588 (51.0)	566 (49.0)	0.83 (0.48, 1.44)	0.95 (0.51, 1.76)
	Others (divorced, widowed)	51 (63.8)	29 (36.2)	1.41 (0.70, 2.85)	0.96 (0.45, 2.05)

Educational status	Unable to read and write	75 (63.0)	44 (37.0)	2.15 (1.36, 3.35)	0.97 (0.58, 1.62)
	Able to read and write	29 (63.0)	17 (37.0)	2.16 (1.13, 4.11)	1.08 (0.53, 2.21)
	Primary (1–8)	292 (57.4)	217 (42.6)	1.70 (1.27, 2.29)	0.88 (0.62, 1.25)
	Secondary (9–12)	228 (44.9)	282 (55.1)	1.02 (0.76, 1.37)	0.68 (0.50, 0.96)
	Diploma and above)	121 (44.2)	153 (55.8)	1	1

Average monthly income	0-1000	202 (51.1)	193 (48.9)	1.77 (1.31, 2.41)	1.72 (1.18, 2.51)
	1001-2000	171 (60.6)	111 (39.4)	2.62 (1.87, 3.66)	2.22 (1.51, 3.27)
	2001-3000	112 (58.0)	81 (42.0)	2.35 (1.62, 3.40)	1.91 (1.26, 2.91)
	3001-4000	89 (58.2)	64 (41.8)	2.36 (1.59, 3.52)	2.03 (1.31, 3.14)
	4001-5000	61 (44.2)	77 (55.8)	1.35 (0.89, 2.03)	1.27 (0.81, 1.99)
	>5000	110 (37.0)	187 (63.0)	1	1

Decisions made on the household issue	By husband	316 (68.1)	148 (31.9)	3.29 (2.59, 4.19)	2.62 (2.01, 3.41)
	By wife	111 (60.0)	74 (40.0)	2.32 (1.67, 3.21)	1.99 (1.33, 2.98)
	Jointly	318 (39.3)	491 (60.7)	1	1

Quarreled with partner's family	Yes	115 (80.4)	28 (19.6)	4.47 (2.91, 6.84)	3.36 (2.14, 5.30)
	No	630 (48.0)	683 (52.0)	1	1

Husband has the right to beat his wife	Yes	60 (61.9)	37 (38.1)	1.60 (1.05, 2.44)	1.36 (0.85, 2.16)
	No	685 (50.3)	676 (49.7)	1	1

Witnessed violence as a child between parents	Yes	318 (68.7)	145 (31.3)	2.92 (2.31, 3.68)	2.34 (1.81, 3.02)
	No	427 (42.9)	568 (57.1)		1

**Table 5:** Partner/nonpartner sociodemographic and behavioral characteristics associated with violence against women in Arsi Zone, 2021.

Variables	Characteristics	VAW during COVID-19			
		Yes (%)	No (%)	COR 95% CI	AOR 95% CI
Age	20-30	265 (50.4)	261 (49.6)	1	1
	31-40	291 (49.3)	299 (50.7)	1.00 (0.76, 1.21)	0.93 (0.72, 1.21)
	41-50	118 (52.9)	105 (47.1)	1.11 (0.81, 1.52)	0.93 (0.65, 1.31)
	>50	71 (59.7)	48 (40.3)	1.46 (0.97, 2.18)	1.19 (0.76, 1.87)

Occupation of your partner/nonpartner	Student	22 (59.5)	15 (40.5)	1	1
	Farmer	115 (65.0)	62 (35.0)	1.27 (0.61, 2.61)	0.68 (0.31, 1.47)
	Employer	267 (46.3)	310 (53.7)	0.59 (0.30, 1.16)	0.40 (0.20, 0.81)
	Merchant	172 (51.7)	161 (48.3)	0.73 (0.37, 1.45)	0.54 (0.26, 1.11)
	Others	169 (50.6)	165 (49.4)	0.70 (0.35, 1.39)	0.47 (0.23, 0.96)

Educational status	Unable to read & write	38 (79.2)	10 (20.8)	4.68 (2.28, 9.60)	2.63 (1.19, 5.81)
	Read and write	32 (61.5)	20 (38.5)	1.97 (1.10, 3.54)	1.05 (0.53, 2.08)
	Primary (1–8)	187 (55.3)	151 (44.7)	1.53 (1.16, 2.01)	0.94 (0.67, 1.32)
	Secondary (9–12)	259 (50.9)	250 (49.1)	1.28 (1.00, 1.63)	0.98 (0.73, 1.31)
	Diploma & above	229 (44.8)	282 (55.2)	1	1

Drink alcohol	Yes	263 (76.7)	80 (23.3)	4.32 (3.27, 5.70)	2.78 (2.10, 3.76)
	No	482 (43.2)	633 (56.8)	1	1

Chew chat	Yes	184 (81.1)	42 (18.9)	5.24 (3.68, 7.46)	3.27 (2.21, 4.85)
	No	561 (45.5)	671 (54.5)	1	1

Smoke cigarette	Yes	69 (90.8)	7 (9.2)	10.3 (4.70, 22.56)	1.80 (0.75, 4.34)
	No	676 (48.9)	706 (51.1)	1	1

Ever fight (aggressively with other men)	Yes	98 (86.0)	16 (14.0)	6.60 (3.85, 11.31)	2.73 (1.51, 4.95)
	No	647 (48.1)	697 (51.9)	1	1

Partner's family involved in decisions	Yes	125 (79.6)	32 (20.4)	4.29 (2.87, 6.42)	2.32 (1.49, 3.62)
	No	620 (47.7)	681 (52.3)	1	1

## Data Availability

The data sets used and/or analyzed during the current study are available from the corresponding author upon reasonable request.

## Abbreviations

AOR: Adjusted odds ratio
COR: Crude odds ratio
COVID-19: Coronavirus disease 19
IPI: Intimate partner violence
NGOs: Nongovernmental organization
NPV: Nonpartner violence
VAW: Violence against women
SDGs: Sustainable Development Goals
UN: United Nations
UNDP: United Nations Development Program
WHO: World Health Organization.
